# Serum Indoxyl Sulfate as a Potential Biomarker of Peripheral Arterial Stiffness in Patients with Non-Dialysis Chronic Kidney Disease Stages 3 to 5

**DOI:** 10.3390/toxins17060283

**Published:** 2025-06-05

**Authors:** Yahn-Bor Chern, Jen-Pi Tsai, Chin-Hung Liu, Yu-Li Lin, Chih-Hsien Wang, Bang-Gee Hsu

**Affiliations:** 1 Division of Nephrology, Department of Internal Medicine, Yuan’s General Hospital, Kaohsiung 80249, Taiwan; culex401@gmail.com; 2 Division of Nephrology, Department of Internal Medicine, Dalin Tzu Chi Hospital, Buddhist Tzu Chi Medical Foundation, Chiayi 62247, Taiwan; tsaininimd1491@gmail.com; 3 School of Medicine, Tzu Chi University, Hualien 97004, Taiwan; nomo8931126@gmail.com; 4 Graduate Institute of Clinical Pharmacy, School of Medicine, Tzu Chi University, Hualien 97004, Taiwan; chinhung@mail.tcu.edu.tw; 5 School of Pharmacy, Tzu Chi University, Hualien 97004, Taiwan; 6 Division of Nephrology, Hualien Tzu Chi Hospital, Buddhist Tzu Chi Medical Foundation, Hualien 97004, Taiwan

**Keywords:** indoxyl sulfate, peripheral arterial stiffness, chronic kidney disease, brachial–ankle pulse wave velocity, cardiovascular risk

## Abstract

Indoxyl sulfate (IS), which is a protein-bound uremic toxin, is involved in vascular dysfunction and cardiovascular risk in subjects with chronic kidney disease (CKD). However, its role in peripheral arterial stiffness (PAS) remains unclear. This cross-sectional study evaluated the relationship between IS and PAS in patients diagnosed with CKD stages 3 through 5 who are not undergoing dialysis. Patients with CKD from a single center were enrolled. High-performance liquid chromatography analyzed the serum IS levels. PAS was evaluated using brachial–ankle pulse wave velocity (baPWV). IS was independently associated with PAS (odds ratio [OR]: 1.389 for 1 μg/mL increase in IS, 95% confidence interval [CI]: 1.086–1.775, *p* = 0.009) in a multivariable analysis after adjustment for age, hypertension, diabetes mellitus, blood pressure, lipid profiles, renal function, albumin, and proteinuria. Moreover, the mean baPWV (*p* = 0.010), left baPWV (*p* = 0.009), and right baPWV (*p* = 0.015) levels significantly correlated with the log-transformed IS (log-IS) levels. The area under the receiver operating characteristic curve for serum IS as a predictor of PAS was determined to be 0.667 (95% CI: 0.580−0.754; *p* = 0.0002). IS was associated with PAS in non-dialysis CKD stages 3–5, suggesting that IS may be a possible vascular risk marker. Future studies should address the nature of the relationship between IS and vascular dysfunction and assess therapeutic strategies to reduce IS.

## 1. Introduction

Chronic kidney disease (CKD) is characterized by a gradual decline in renal function, leading to the accumulation of uremic toxins that result in systemic complications. Despite advancements in detecting and treating the risk factors of CKD, its prevalence is approximately 10% and ranges from 11% to 14% [1,2]. Cardiovascular disease (CVD), which is the leading cause of morbidity and mortality in patients with CKD, is a significant concern [3]. As the Kidney Disease Improving Global Outcomes (KDIGOs) guidelines have recommended considering all patients with CKD as high-risk for CVD, arterial stiffness (AS), which is a critical component of CV manifestations, has been considered a significant predictor of CVD and all-cause mortality as well as responsible for the onset and deterioration of kidney disease in these patients [4].

AS is a vasculopathy that is characterized by reduced elasticity and increased rigidity of the arterial walls due to aging and arteriosclerosis [5,6,7,8]. Clinically, two techniques use pulse wave velocity (PWV) to measure AS: carotid–femoral PWV (cfPWV) and brachial–ankle PWV (baPWV) [6,7,8]. Both PWV methods accurately predict CV outcomes and CKD progression with similar efficacy [9,10,11]. Although cfPWV is a good standard for evaluating central AS, baPWV has some practical advantages over cfPWV. BaPWV is less operator-dependent and can be measured more easily and quickly due to its automated nature and ease of use when applied to oscillometric devices [6,7,8]. Additionally, baPWV measures peripheral artery elasticity and reflects peripheral AS (PAS), allowing for a comprehensive evaluation of overall vascular health in patients with CKD [6,7,8]. A comparison between the two methods is shown in Table 1 [6,7,8].

The prognostic ability of baPWV for CV events and mortality has been consistently demonstrated in various populations. Ohkuma et al. showed in a meta-analysis that baPWV predicts future CV events with a high significance level and revealed this association through an analysis of individual participant data in 14,673 Japanese people with no history of CVD [12]. Specifically, they found that the highest quintile of baPWV had a 3.50-fold risk for incident CVD compared to the lowest; furthermore, each one standard deviation of baPWV was associated with a 1.19-fold risk for CVD incidence. Adding baPWV to the Framingham risk score significantly enhanced its statistical significance in predicting CVD, showing the linear dose response of baPWV and an underlying elevated vascular risk. Moreover, increased baPWV predisposes groups at risk for increased all-cause and CV mortality. For instance, in patients with type 2 diabetes, the highest quartile of baPWV predicted an independent 63% increase in all-cause mortality and a 75% increase in expanded CVD mortality [13]. Similarly, in acute stroke, the group with the highest tertile of baPWV had a 97% increase in all-cause death and a 139% increase in vascular death [14]. Thus, increased AS is critical for long-term morbidity in chronic states.

Indoxyl sulfate (IS) is a protein-bound uremic toxin produced by the metabolism of tryptophan by intestinal bacteria. Due to impaired renal clearance, IS levels are significantly elevated in patients with CKD [15]. Although a meta-analysis failed to establish a direct association between increased IS levels and poor CV outcomes [16], one study reported that IS was associated with early left ventricular systolic dysfunction [17]. Regarding the biochemical actions of IS, it acts as a predictor of deterioration in renal function [17] and promotes endothelial dysfunction [18,19], vascular calcification [20,21], oxidative stress, and inflammation [22], all of which could potentially increase the risk for AS. This makes IS a promising vascular biomarker for predicting AS in patients with CKD.

In our previous study, IS predicted central AS as measured by cfPWV [23]. Given the advantages and the predictive role of baPWV, the relationship between IS and baPWV in patients with CKD requires further investigation. This study aims to investigate the association between levels of IS and PAS in stages 3–5 CKD patients who are not on dialysis, based on baPWV. The identification of such a relationship may help characterize the role of baPWV as an easy-to-administer variable in the evaluation of PAS and establish the feasibility of implementing IS data into a more holistic approach to vascular health assessment through tandem monitoring of this variable and baPWV in such patients. As a result, use of this information may further facilitate the refinement of patient stratification in current or future clinical algorithms aimed at reducing the incidence of CVD-related morbidity and mortality in this population.

## 2. Results

### 2.1. Patient Characteristics

Table 2 presents the clinical characteristics of the 146 patients with non-dialysis CKD stages 3–5 included in this study. Overall, 62 (42.47%) patients had PAS. The PAS group had a significantly older mean age than the control patients (73.42 ± 11.97 years vs. 64.74 ± 11.89 years, *p* < 0.001). Additionally, those with PAS had a significantly higher systolic blood pressure (SBP, *p* < 0.001), diastolic blood pressure (DBP, *p* = 0.013), and serum creatinine level (*p* = 0.044), but a lower estimated glomerular filtration rate (eGFR) (*p* = 0.003). The patients with PAS tended to have more advanced CKD stages (*p* = 0.037). Among the patients with PAS, serum IS levels (*p* = 0.001) as well as the prevalence of diabetes mellitus (DM, 50.0% vs. 32.1%, *p* = 0.029) and hypertension (74.2% vs. 50.0%, *p* = 0.003) were higher when compared with the control patients. Patients with PAS also had a significantly higher urine protein-to-creatinine ratio (UPCR, *p* = 0.016) and lower serum albumin levels (*p* = 0.013) than the patients without PAS. However, there were no significant differences in body mass index (BMI), hemoglobin (Hb) levels, lipid profile levels, and use of anti-hypertensives or lipid-lowering medications.

### 2.2. Identification of Clinical Factors Associated with PAS

We conducted a multivariable logistic regression analysis to determine which factors were independently associated with PAS. As presented in Table 3, PAS is significantly associated with increased serum IS concentrations (odds ratio [OR]: 1.389 for every 1 μg/mL increase in IS, 95% confidence interval [CI]: 1.086–1.775, *p* = 0.009) after adjusting for potential confounding factors such as DM, hypertension, age, SBP, DBP, albumin, creatinine, eGFR, fasting glucose, total cholesterol, triglyceride, low-density lipoprotein cholesterol, and UPCR. Age was also a significant factor, with each additional year conferring increased odds of PAS (OR: 1.125, 95% CI: 1.060–1.188, *p* < 0.0001). Moreover, DM and a higher DBP were also significantly associated with PAS (OR: 5.111, 95% CI: 1.612–16.201, *p* = 0.006 and OR: 1.091, 95% CI: 1.022–1.165, *p* = 0.009, respectively).

### 2.3. Evaluating the Diagnostic Power of Serum IS for PAS

**Figure 1:**
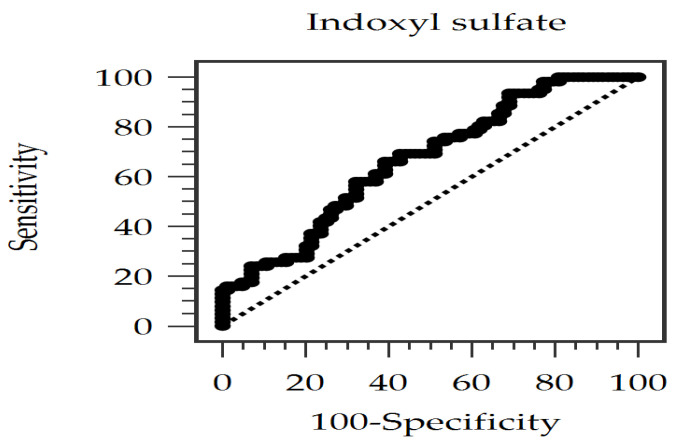
The area under the receiver operating characteristic curve signifies the diagnostic efficacy of indoxyl sulfate concentrations for predicting peripheral arterial stiffness.

The receiver operating characteristic (ROC) curve was drawn and presented to assess the diagnostic potential for PAS according to concentrations of IS (Figure 1). The area under the ROC curve (AUC) was calculated as 0.667 (95% CI: 0.580−0.754; *p* = 0.0002), which is compatible with a moderate discrimination ability. The best cut-off level of IS was identified as 0.996 μg/mL, with a sensitivity of 66.13%, a specificity of 60.71%, a positive predictive value of 55.41%, and a negative predictive value of 70.83%. This suggests that elevated IS levels may be a novel diagnostic marker for identifying patients with CKD at high risk for PAS, but it may serve as an adjunct, not an isolated diagnostic measure, because of its moderate diagnostic ability.

### 2.4. Analyses of the Correlation

In the Spearman correlation analysis (Table 4), the factors that were positively and significantly correlated with the mean baPWVs included age (*p* < 0.001), SBP (*p* < 0.001), and DBP (*p* < 0.001), emphasizing the role of aging and hemodynamic factors in the development of AS. Log-transformed IS (log-IS) had a significant, positive correlation with the mean baPWVs (*p* = 0.010), indicating a possible contribution to the development of PAS. The other correlations related to log-IS included positive correlations with log-transformed blood urea nitrogen (log-BUN, *p* < 0.001) and log-creatinine (*p* < 0.001), which are solutes related to renal dysfunction, and a negative correlation with albumin (*p* = 0.004) and Hb (*p* = 0.003), suggesting their role in CKD-related metabolic and vascular disturbances. In contrast, eGFR was inversely correlated with the mean baPWVs (*p* = 0.015) as well as with log-IS (*p* < 0.001), indicating that declining renal function is associated with both higher IS levels and increased PAS. For readers interested in assessing finer resolutions, the separate correlation results for left and right baPWVs were made available in a supplementary file (Supplementary Table S1).

## 3. Discussion

The multivariable logistic regression analyses revealed that an elevated IS, a higher DBP, advanced age, and DM were independent risk factors for PAS in patients with non-dialysis CKD stages 3–5. IS demonstrated moderate discriminating power for PAS (AUC = 0.667). Furthermore, log-IS correlated positively with bilateral baPWV, log-BUN, and log-creatinine, and negatively with albumin, Hb, and eGFR.

Our results reinforce the role of conventional CV risk factors in PAS. DM and PAS host common pathologic pathways, such as endothelial dysfunction, that hyperglycemia can aggravate [24,25,26]. Hypertension is a known predictor, promoting PAS due to mechanical stress and endothelial damage [27,28,29] and possibly leading to a self-perpetuating situation along with the stiffening of arteries. Older age is a recognized ‘driver’ of vascular dysregulation through structural changes to the arteries [5,6,7,30] and through age-associated anthropometric changes [31], emphasizing the need to address these modifiable risk factors. As reported in previous works [6,7,11], baPWVs were positively correlated with a decreased eGFR. The relationship between impaired renal function and vascular stiffness may be due to CKD-related chronic inflammation, hemodynamic changes, increased oxidative stress, uremic toxin [32,33], CKD-mineral bone disease, and vascular calcification [6,7,34]. The association between proteinuria and AS is likely to be bidirectional; that is, proteinuria may represent profound renal and endothelial damage, and devastated microcirculation and stiff arteries may be an aggravating factor of renal damage [11,35]. Therefore, a timely assessment of proteinuria is essential to addressing it as a risk factor and therapeutic target to ameliorate CV outcomes in CKD [35].

The pathogenic role of IS in inducing inflammation, oxidative stress, and vascular calcification [19,36,37,38]. The observed correlations of IS with SBP, BUN, creatinine, and UPCR (positive), and with Hb, albumin, and eGFR (negative) reflect an interconnected pathophysiology [39]. Elevated IS levels in renal dysfunction patients are primarily due to impaired renal clearance. IS can induce renal tubular and endothelial cell damage and inflammation, promoting CKD progression and increasing UPCR [20,40,41]; conversely, proteinuria signifies advanced renal injury that would also lead to reduced IS clearance. IS can promote vascular calcification, inflammation, and oxidative stress, leading to vascular remodeling and increased BP [16,20,36], while hypertension can, in turn, impair IS clearance due to hypertension-related renal damage [17,22]. The negative IS-Hb correlation might be partly explained by IS-induced inflammation or reduced antioxidant enzyme activity in red blood cells (RBCs), leading to RBC fragility [38]. The inverse IS–albumin correlation could be due to proteinuria-led albumin loss; additionally, low albumin levels or uremia-induced structural modifications of albumin might impair the clearance of protein-bound IS, increasing circulating free IS [40,41]. The latter associations, especially Hb and albumin, warrant further clarification.

As shown in Table 5, a remarkable phenomenon is present in that most biomarkers with excellent diagnostic predictive value for AS or CV events (larger AUCs) were applied in ESRD patients. In contrast, the diagnosis of PAS in patients with non-dialysis CKD stages 3–5, including those in the present study, is, in general, more difficult, as the interplay of pathomechanisms is complex and variable as described before. Due to its complexity and the limited diagnostic performance of IS testing in this particular group of persons, a more stringent diagnostic algorithm is needed. Thus, for non-dialysis CKD patients, the integration of IS with traditional clinical variables—including age, blood pressure, diabetic status, and indicators of renal function, which were also confirmed by the present multivariate analyses as determinants of PAS—seems to warrant a more holistic and accurate evaluation of PAS, which is also consistent with the concept of IS being an adjunctive rather than exclusive PAS diagnostic means.

The main finding in the present study may be the possibility of using IS as a novel biomarker for recognizing PAS susceptibility in non-dialysis CKD stages 3–5. Since AS is a significant risk factor for all-cause and CV mortality, IS measurement together with baPWV may be added as a mode for early participant vascular risk stratification. Although IS had only moderate discrimination and may act as an ancillary rather than individual diagnostic test, it represents a useful tool to evaluate vascular health status in CKD, particularly in patients with the concomitant comorbidities of hypertension and DM. The strong associations of PAS with modifiable factors such as BP and proteinuria mean attention must be paid to their aggressive control. As IS accumulates due to decreased renal function, therapies that decrease IS levels, including changes in diet and therapies that modulate the composition of the gut microbiome, may represent attractive means for lowering vascular disease in CKD patients [48].

There are several limitations to this study. First, a causal relationship between IS concentrations and the progress of PAS cannot be ascertained due to the cross-sectional design; thus, longitudinal studies with stratification by individual CKD stage to delineate stage-specific IS effects on AS are necessary, considering the heterogeneity brought by different CKD stages. Second, despite the corrections applied, the residual confounding associated with the complex cascade of metabolic, hemodynamic, and inflammatory drivers of vascular disturbance in CKD cannot be wholly excluded. Third, we might not be able to extrapolate our results to other populations, given that all the patients were recruited from a single center. Fourth, we measured total IS levels only; however, an analysis of free IS levels in future studies is of interest to clarify the clinical implications of IS. Interventional trials studying IS-reducing strategies for CV protection and mechanistic studies of the molecular pathomechanisms by which IS is linked to vascular dysfunction would be warranted.

## 4. Conclusions

In conclusion, we determined that IS was an independent related factor of PAS in patients with non-dialysis CKD stages 3–5, suggesting its potential role as a vascular biomarker in this population. Additionally, higher IS levels and conventional CV risk factors, including advanced age, hypertension, and DM, were also significantly associated with PAS development. Employing the baPWV may provide patients with CKD an opportunity for early vascular risk stratification, contributing to the early management of AS and reducing the risk of CV complications. Longitudinal and interventional studies are needed to validate the prognostic utility of IS for PAS and to confirm if strategies that aim to lower IS levels can improve vascular health and overall CV outcomes, specifically in patients with CKD. Moreover, mechanistic studies identifying the pathways mediating IS and AS may assist in defining the potential opportunities and strategies for therapeutic intervention.

## 5. Materials and Methods

### 5.1. Enrollment of Patients and Collection of Data

From January 2020 to December 2020, 146 patients from the outpatient clinics of the renal department at a medical center in Hualien, Taiwan were included in this cross-sectional study. To be included in this study, the patients needed to be adults (no less than eighteen years old) with stages 3 to 5 of CKD who did not require maintenance dialysis. CKD was defined as a CKD-Epidemiology Collaboration eGFR of <60 mL/min/1.73 m^2^ on two separate evaluations with an interval of at least 3 months apart. CKD staging was categorized based on the Kidney Disease Outcomes Quality Initiative criteria as follows: CKD stage 3, eGFR 30–59 mL/min per 1.73 m^2^; CKD stage 4, eGFR 15–29 mL/min per 1.73 m^2^; and CKD stage 5, eGFR < 15 mL/min per 1.73 m^2^. Patients with the following conditions were excluded: malignancy; current infections; recent acute coronary events; acute decompensated heart failure; cerebrovascular accidents; a documented history of chronic inflammatory disorders, including inflammatory bowel disease or rheumatoid arthritis; chronic obstructive pulmonary disease; those on maintenance dialysis; and those who did not provide consent. The study protocol was approved by the Research Ethics Committee of Hualien Tzu Chi Hospital, Buddhist Tzu Chi Medical Foundation (approval number: IRB108-219-A). Written informed consent was obtained from each patient before the enrollment process.

Each patient’s basic demographic and biochemical variables were obtained from their electronic medical records, including age, sex, etiology of CKD, and medications for treating hypertension or dyslipidemia. BP was obtained by asking a participant to sit for 10 min in a quiet and comfortable environment; then, trained staff measured their BP using a sphygmomanometer with mercury that was calibrated and a cuff of the correct size. Hypertension was confirmed when a patient’s SBP was not less than 140 mm Hg, their DBP was not less than 90 mm Hg, or the patient had already taken blood pressure-lowering agents within 2 weeks of their participation. The diagnosis of DM was based on a fasting glycemic concentration ≥ 126 mg/dL or if a patient was on antidiabetic medications.

### 5.2. Anthropometric Details and Serum Samples Assessment (Biochemical and Total IS Determination)

BMI was obtained by dividing a patient’s body weight (kg) by the square of their height (m^2^). Following an overnight fast, approximately 5 mL of blood was obtained from each participant; 0.5 mL of this sample was used to measure Hb levels using the Sysmex SP-1000i (Sysmex America, Mundelein, IL, USA). The residual 4.5 mL was centrifuged at 3000× *g* for a duration of 10 min and assessed by an autoanalyzer (Siemens Advia 1800; Siemens Healthcare, Henkestr, Erlangen, Germany) to quantify the serum concentrations of fasting glucose, total cholesterol levels, triglycerides, BUN, creatinine, albumin, low-density lipoprotein cholesterol, and spot UPCR.

To measure serum total IS concentrations, liquid chromatography-mass spectrometry analysis with a modified protocol with the Waters e2695 high-performance liquid chromatography system (ACQUITY QDa, Waters Corporation, Milford, MA, USA) was employed, as described previously [18,23]. The mass spectrometer was operated in both positive and negative ion modes across comprehensive scan ranges of 50–450 *m*/*z* and 100–350 *m*/*z*, respectively, to monitor the distinct compound (IS: 211.9 *m*/*z*). All analyses were conducted utilizing the Empower^®^ 3.0 software (New York, NY, USA).

### 5.3. Using baPWV for the Detection of PAS

BaPWV values were measured for diagnosing PAS by measuring an arterial segment’s length extending from the brachium to the ankle and dividing it by the time interval between the brachial and ankle wavefronts. Properly trained staff were responsible for all the measurements of baPWVs (VP-2000, Omron, Kyoto, Japan). The proposed cut-off value for diagnosing PAS was determined by referencing the Physiological Diagnosis Criteria for Vascular Failure Committee of Japan, as studies regarding this topic were mainly conducted in Japan. A baPWV value greater than eighteen meters per second was deemed statistically sufficient as a cut-off for individuals presenting a heightened risk of cardiovascular disease and those who are hypertensive [8,49]. Therefore, in this study, we categorized patients whose baPWV value of either side was greater than eighteen meters per second into the PAS cohort.

### 5.4. Statistical Analyses

This study performed a power analysis to identify the minimum sample size to reveal a significant correlation between IS levels and PAS. A minimum clinically meaningful correlation coefficient (r ≥ 0.25) was chosen to balance between Cohen’s recommendations for moderate effect size and previous studies that associated uremic toxins with vascular dysfunction in CKD [23,27,28]. At α = 0.05 and power = 80%, the required sample size was 123. With *n* = 146, the effect size achieved at >90% power at α = 0.05 supports reliable detection and reflects clinically relevant IS consequences on vascular health.

We assessed all variables for adherence to normality prior to conducting the linear regression analyses. We employed the Kolmogorov–Smirnov test to assess the normality of continuous variables. Normally distributed variables were reported as mean ± standard deviation, and a comparison was made between the PAS and non-PAS groups using the unpaired Student’s *t*-test. Meanwhile, the Mann–Whitney U test was used to assess the parameters that did not exhibit normality. The chi-square test presents categorical values in numbers and percentages and compares them between groups. The clinical parameters that were significantly different between the groups and the unknown risk factors of cardiovascular diseases published in previous studies (i.e., fasting glucose, total cholesterol, triglyceride, and low-density lipoprotein cholesterol) were regarded as adjustable variables and incorporated into a multivariable logistic regression model to ascertain independent risk factors for PAS.

Parameters with skewed distributions (i.e., IS, triglyceride, glucose, BUN, creatinine, and UPCR) were logarithmically transformed (log) before the correlation analyses. Spearman correlation analysis was used to determine the log-IS concentrations and the correlation of bilateral baPWV values with other continuous variables. An ROC curve analysis was conducted to further explore the correlation of serum IS levels with PAS. All statistical analyses were conducted using IBM SPSS Statistics for Windows, version 19.0 (IBM Corp., Armonk, NY, USA).

## Figures and Tables

**Table 1:** Summary of key differences between carotid–femoral pulse wave velocity and brachial–ankle pulse wave velocity.

Aspect	Carotid–Femoral PWV (cfPWV)	Brachial–Ankle PWV (baPWV)
Measurement Technique	Using tonometric devices, focusing on central arteries (elastic arteries).	Using oscillometric devices, assessing both central and peripheral arteries (elastic and muscular arteries).
Reproducibility	Highly operator-dependent but reliable after proper training.	Highly reproducible due to the automated nature of the measurement.
Clinical Relevance	Gold standard for central AS but time-consuming; associated with renal and CV outcomes.	Simpler and faster to perform; correlates with renal and CV outcomes.
Patient Considerations	Less affected by PAD; preferred for assessment of aortic stiffness.	Less reliable in PAD; useful for overall vascular health assessment.

**Table 2:** The clinical parameters observed in patients with chronic kidney disease, categorized by the presence or absence of peripheral arterial stiffness.

Parameters	All Patients (*n* = 146)	Non-PAS Group (*n* = 84)	PAS Group (*n* = 62)	*p* Value
Age (years)	68.42 ± 12.64	64.74 ± 11.89	73.42 ± 11.97	<0.001 *
Height (cm)	159.49 ± 8.99	159.92 ± 9.73	158.90 ± 7.91	0.496
Body weight (kg)	66.55 ± 12.45	68.08 ± 14.12	64.47 ± 9.47	0.084
Body mass index (kg per m^2^)	26.06 ± 3.80	26.48 ± 4.34	25.49 ± 2.85	0.120
Left baPWV (m/s)	17.31 ± 3.68	14.85 ± 1.54	20.66 ± 3.05	<0.001 *
Right baPWV (m/s)	17.27 ± 3.47	14.94 ± 1.97	20.42 ± 2.41	<0.001 *
SBP (mmHg)	149.61 ± 26.00	142.07 ± 22.57	159.82 ± 27.02	<0.001 *
DBP (mmHg)	83.97 ± 13.75	81.55 ± 12.13	87.24 ± 15.16	0.013 *
Total cholesterol (mg/dL)	162.29 ± 43.73	162.48 ± 47.26	162.03 ± 38.81	0.952
Triglyceride (mg/dL)	120.50 (86.00–168.25)	114.50 (83.00–167.75)	132.50 (86.75–171.75)	0.360
LDL-C (mg/dL)	91.46 ± 35.99	92.89 ± 39.72	89.52 ± 30.41	0.577
Fasting glucose (mg/dL)	111.00 (97.75–138.25)	109.50 (97.00–123.50)	117.00 (100.50–144.00)	0.132
Blood urea nitrogen (mg/dL)	32.00 (24.75–44.00)	29.50 (24.00–43.00)	34.50 (26.00–50.75)	0.154
Creatinine (mg/dL)	1.80 (1.40–2.63)	1.70 (1.40–2.50)	2.05 (1.60–3.13)	0.044 *
eGFR (mL/min)	32.03 ± 15.28	35.20 ± 14.90	27.72 ± 14.84	0.003 *
Spot UPCR (g/g)	0.47 (0.17–1.20)	0.35 (0.14–0.96)	0.61 (0.21–1.92)	0.016 *
Albumin (g/dL)	4.07 ± 0.39	4.14 ± 0.38	3.98 ± 0.39	0.013 *
Hemoglobin (g/dL)	11.66 ± 2.64	11.74 ± 2.07	11.55 ± 3.28	0.682
Indoxyl sulfate (μg/mL)	1.05 (0.55–2.32)	0.80 (0.41–1.74)	1.45 (0.70–3.62)	0.001 *
Female, *n* (%)	68 (46.6)	37 (44.0)	31 (50.0)	0.476
Diabetes mellitus, *n* (%)	58 (39.7)	27 (32.1)	31 (50.0)	0.029 *
Hypertension, *n* (%)	88 (60.3)	42 (50.0)	46 (74.2)	0.003 *
Glomerulonephritis, *n* (%)	34 (23.3)	21 (25.0)	13 (21.0)	0.569
ARB use, *n* (%)	76 (52.1)	40 (47.6)	36 (58.1)	0.212
β-blocker use, *n* (%)	31 (21.2)	17 (20.2)	14 (22.6)	0.732
CCB use, *n* (%)	60 (41.1)	31 (36.9)	29 (46.8)	0.231
α-adrenergic blocker, *n* (%)	22 (15.1)	10 (11.9)	12 (19.4)	0.214
Statin use, *n* (%)	67 (45.9)	36 (42.9)	31 (50.0)	0.392
Fibrate use, *n* (%)	26 (17.8)	16 (19.0)	10 (16.1)	0.649
CKD stage 3, *n* (%)	76 (52.1)	51 (60.7)	25 (40.3)	0.037 *
CKD stage 4, *n* (%)	41 (28.1)	21 (25.0)	20 (32.3)	
CKD stage 5, *n* (%)	29 (19.8)	12 (14.3)	17 (27.4)	

Continuous variables are expressed as mean ± standard deviation and compared by Student’s *t*-test, or median and interquartile range, and compared using the Mann–Whitney *U* test for those that fail to conform to a normal distribution, whereas categorical variables are expressed as a number (%) and compared by a chi-square test. PAS refers to peripheral arterial stiffness; baPWV denotes brachial–ankle pulse wave velocity. SBP indicates systolic blood pressure; DBP signifies diastolic blood pressure. CKD represents chronic kidney disease; LDL-C stands for low-density lipoprotein cholesterol. eGFR refers to estimated glomerular filtration rate; UPCR denotes urine protein-to-creatinine ratio. ARB signifies angiotensin-receptor blockade agents; CCB represents calcium-channel blocker. * *p* < 0.05 was deemed statistically significant.

**Table 3:** Multivariable logistic regression analysis pertaining to the determinants associated with peripheral arterial stiffness.

Determinants	Odds Ratio	95% Confidence Interval	*p* Value
Indoxyl sulfate, 1 μg/mL	1.389	1.086–1.775	0.009 *
Age, 1 year	1.125	1.060–1.188	<0.001 *
Presence of diabetes mellitus	5.111	1.612–16.201	0.006 *
Diastolic blood pressure, 1 mmHg	1.091	1.022–1.1665	0.009 *
Systolic blood pressure, 1 mmHg	0.985	0.946–1.026	0.468
Hypertension, present	1.341	0.383–4.696	0.646
Albumin, 1 g/dL	0.911	0.215–3.859	0.899
Creatinine, 1 mg/dL	0.503	0.241–1.050	0.067
eGFR, 1 mL/min	0.968	0.917–1.022	0.245
Spot UPCR, 1 g/g	1.467	0.863–2.492	0.157
Fasting glucose, 1 mg/dL	0.990	0.978–1.002	0.102
Total cholesterol, 1 mg/dL	1.019	0.995–1.044	0.115
Triglyceride, 1 mg/dL	1.000	0.996–1.003	0.781
LDL-C (mg/dL)	0.976	0.949–1.004	0.090

Clinical determinants were adjusted using multivariable logistic regression analysis with factors adopted such as diabetes mellitus, hypertension, age, systolic blood pressure, diastolic blood pressure, albumin, creatinine, eGFR, fasting glucose, total cholesterol, triglyceride, LDL-C, spot UPCR, and indoxyl sulfate. LDL-C denotes low-density lipoprotein cholesterol; eGFR represents estimated glomerular filtration rate and UPCR refers to the urine protein-to-creatinine ratio. * *p* < 0.05 was deemed statistically significant.

**Table 4:** Spearman correlation coefficients between mean baPWV, log-IS, and clinical variables.

Variables	Mean baPWV (m/s)		Log-IS (μg/mL)	
	Spearman’s Correlation Coefficient	*p* Value	Spearman’s Correlation Coefficient	*p* Value
Age (years)	0.379	<0.001 *	0.076	0.363
Body mass index (kg/m^2^)	−0.093	0.265	−0.076	0.363
Mean baPWV (m/s)	—	—	0.213	0.010 *
Log-IS (μg/mL)	0.213	0.010 *	—	—
Systolic BP (mmHg)	0.371	<0.001 *	0.310	<0.001 *
Diastolic BP (mmHg)	0.295	<0.001 *	0.125	0.133
Total cholesterol (mg/dL)	0.030	0.717	−0.140	0.092
Log-Triglyceride (mg/dL)	0.017	0.838	0.001	0.995
LDL-C (mg/dL)	0.056	0.501	−0.131	0.114
Log-Glucose (mg/dL)	0.061	0.465	0.134	0.106
Albumin (mg/dL)	−0.132	0.112	−0.239	0.004 *
Hemoglobin (g/dL)	0.085	0.307	−0.241	0.003 *
Log-BUN (mg/dL)	0.096	0.249	0.534	<0.001 *
Log-Creatinine (mg/dL)	0.155	0.062	0.607	<0.001 *
eGFR (mL/min)	−0.201	0.015 *	−0.548	<0.001 *
Log-UPCR (g/g)	0.202	0.014 *	0.385	<0.001 *

Data were analyzed using Spearman correlation analysis. The mean baPWV column represents the average of the left and right brachial–ankle pulse wave velocity (baPWV) values, calculated as (left baPWV + right baPWV)/2. Variables such as IS, triglyceride, glucose, BUN, creatinine, and UPCR exhibited a skewed distribution, necessitating their logarithmic transformation prior to conducting the analysis. IS, indoxyl sulfate; baPWV, brachial–ankle pulse wave velocity; BP, blood pressure; LDL-C, low-density lipoprotein cholesterol; BUN, blood urea nitrogen; eGFR, estimated glomerular filtration rate; UPCR, urine protein-to-creatinine ratio. * *p* < 0.05 was considered statistically significant.

**Table 5:** Summary of the literature on AUC values for serum markers diagnosing arterial stiffness or related vascular conditions in CKD patients.

Marker	Population Studied	AS/Outcome Measured	Reported AUC	Sensitivity/Specificity (at Optimal Cutoff, If Available)	Citation
IS	Non-dialysis CKD stages 3–5	PAS (baPWV > 18 m/s)	0.667	66.13%/60.71% (at 0.996 µg/mL)	Current Study
Sclerostin	ESRD	Aortic stiffness (cfPWV > 10 m/s)	0.673	35.14%/91.67% (at 208.64 pmol/L)	[42]
PCS	Non-dialysis CKD stages 3–5	PAS (baPWV > 18 m/s)	0.628	53.7%/70.9% (at 20.49 mg/L)	[43]
B2M	CKD stage ≥ 3, including dialysis	CV event	0.684	85.1%/52.1% (at 4.21 mg/dL	[44]
Cr	CKD stage ≥ 3, including dialysis	CV event	0.563	Not reported	[44]
OPN	PD	Aortic stiffness (cfPWV > 10 m/s)	0.903	86.36%/91.67% (at 39.67 ng/mL	[45]
MDA-LDL	HD	PAS (baPWV > 18 m/s)	0.717	79.25%/59.57% (at 80.91 mg/dL	[46]
suPAR	HD	Aortic stiffness (cfPWV > 10 m/s)	0.81	60.0%/88.7% (at 4.85 pg/mL)	[47]

AS is an abbreviation for arterial stiffness; AUC represents the area under the receiver operating characteristic curve. baPWV denotes brachial–ankle pulse wave velocity; B2M refers to beta-2-microglobulin. cfPWV indicates carotid–femoral pulse wave velocity; CI stands for confidence interval. CKD signifies chronic kidney disease; Cr is an abbreviation for creatinine. CV relates to cardiovascular; ESRD represents end-stage renal disease. HD indicates hemodialysis; IS refers to indoxyl sulfate. MDA-LDL signifies malondialdehyde-modified low-density lipoprotein; OPN stands for osteopontin. PAS denotes peripheral arterial stiffness; PCS represents p-cresyl sulfate. PD indicates peritoneal dialysis; Sens is a term for sensitivity. Spec refers to specificity; suPAR signifies soluble urokinase-type plasminogen activator receptor.

## Data Availability

The original contributions presented in this study are included in this article and supplementary material. Further inquiries can be directed to the corresponding author.

## Supplementary Materials

The following supporting information can be downloaded at https://www.mdpi.com/article/10.3390/toxins17060283/s1: Table S1: Spearman correlation coefficients between left baPWV, right baPWV, log-IS, and clinical variables.

## Abbreviations

ARICs	Atherosclerosis risk in communities
AS	Arterial stiffness
AUC	Area under the ROC curve
BMI	Body mass index
CKD	Chronic kidney disease
DM	Diabetes mellitus
IS	Indoxyl sulfate
KDIGOs	Kidney Disease Improving Global Outcomes
PAS	Peripheral arterial stiffness
PCS	P-cresyl sulfate
PWV	Pulse wave velocity
RBCs	Red blood cells
ROC	Receiver operating characteristic
UPCR	Urine protein-to-creatinine ratio
