# Supplementary material for: Serum Indoxyl Sulfate as a Potential Biomarker of Peripheral Arterial Stiffness in Patients with Non-Dialysis Chronic Kidney Disease Stages 3 to 5
Source: Toxins (Basel). 2025 Jun 5;17(6):283. doi: 10.3390/toxins17060283 (PMC12197566; doi:10.3390/toxins17060283)
Supplement: Supplementary file 1 [file toxins-17-00283-s001.zip › toxins-3638777-supplementary.pdf]

Table S1. Spearman correlation coefficients between left baPWV, right baPWV, log-IS, and clinical variables in 146 chronic kidney disease patients.

**Variables**	Left baPWV (m/s)		Right baPWV (m/s)		Log-IS (μg/mL)	
	Spearman coefficient of correlation	*p* value	Spearman coefficient of correlation	*p* value	Spearman coefficient of correlation	*p* value
Age (years)	0.374	<0.001*	0.368	<0.001*	0.076	0.363
Body mass index (kg/m^2)	–0.068	0.362	–0.107	0.200	–0.076	0.363
Left baPWV (m/s)	—	—	0.914	<0.001*	0.215	0.009*
Right baPWV (m/s)	0.914	<0.001*	—	—	0.201	0.015*
Log-IS (μg/mL)	0.215	0.009*	0.201	0.015*	—	—
SBP (mmHg)	0.393	<0.001*	0.332	<0.001*	0.310	<0.001*
DBP (mmHg)	0.307	<0.001*	0.269	0.001*	0.125	0.133
Total cholesterol (mg/dL)	0.004	0.960	0.057	0.497	–0.140	0.092
Log-Triglyceride (mg/dL)	0.009	0.915	0.025	0.764	0.001	0.995
LDL-C (mg/dL)	0.040	0.630	0.070	0.398	–0.131	0.114
Log-Glucose (mg/dL)	0.054	0.521	0.066	0.428	0.134	0.106
Albumin (mg/dL)	–0.149	0.072	0.108	0.194	–0.239	0.004*
Hemoglobin (g/dL)	0.071	0.397	0.097	0.245	–0.241	0.003*
Log-BUN (mg/dL)	0.084	0.312	0.104	0.211	0.534	<0.001*
Log-Creatinine (mg/dL)	0.152	0.067	0.151	0.068	0.607	<0.001*
eGFR (mL/min)	–0.199	0.016*	–0.195	0.019*	–0.548	<0.001*
Log-UPCR (g/g)	0.200	0.016*	0.196	0.018*	0.385	<0.001*

Data were analyzed using Spearman correlation analysis. IS, triglyceride, glucose, BUN, creatinine, and UPCR were skewed distribution data and were log-transformed before analysis. IS, indoxyl sulfate; baPWV, brachial-ankle pulse wave velocity; SBP, systolic blood pressure; DBP, diastolic blood pressure; LDL-C, low-density lipoprotein cholesterol; BUN, blood urea nitrogen; eGFR, estimated glomerular filtration rate; UPCR, urine protein-to-creatinine ratio. *$p < 0.05$ was considered statistically significant.
